# A novel approach to micro-fabricated thermoelectric generators with SrTiO_3_

**DOI:** 10.1080/14686996.2026.2665920

**Published:** 2026-05-08

**Authors:** Joaquin Santander, Iñigo Martin-Fernandez, Carlos Carbonell, Alex Rodríguez-Iglesias, Laura Fuentes-Rodríguez, Marta Fernández-Regúlez, Llibertat Abad, Aitor F. Lopeandia, Arindom Chatterjee, Nini Pryds, Luis Fonseca, Marc Salleras

**Affiliations:** a Micro and Nano Systems Department, Institute of Microelectronics of Barcelona (IMB-CNM-CSIC), Bellaterra, Spain; b Physics Department, Universitat Autònoma de Barcelona, Bellaterra, Spain; c GTNAM at Institut Català de Nanociència i Nanotecnologia (ICN2), Bellaterra, Spain; d Department of Energy Conversion and Storage, Technical University of Denmark, Lyngby, Denmark

**Keywords:** Thermoelectric, micro-thermoelectric generator, thin-films, SrTiO_3_, IoT

## Abstract

The growing demand for autonomous, sustainable, and delocalized power sources for low-power-consuming electronic devices is driving a significant research effort on energy harvesting technologies. Among these, micro-thermoelectric generators (µTEGs) emerge as an appealing solution because of the abundance of residual latent heat sources. This paper proposes an approach to combine high-performance thermoelectric oxides with Si-based µTEGs, leveraging the miniaturization and high-density integration of CMOS-like technologies. The approach is applied to the integration of niobium-doped strontium titanate (Nb:STO) thin films on Si-based planar µTEG structures. The Nb:STO-based µTEG achieves a specific power density Γ = 0.36 nW·cm^−2^·K^−2^ under controlled temperature gradients, which is below state-of-the-art performance probably due to lower electrical conductivity from polycrystalline growth. When the chips were tested under realistic operating conditions – placed on a hot surface at 175°C – a maximum power output of *p* = 0.07 nW was obtained. Nonetheless, by implementing a technological solution for thermal dissipation, the temperature gradient across the thermoelectric material improved by a factor of 110, resulting in a significantly higher extracted power of *p* = 7.75 nW.

## IMPACT STATEMENT

We present a versatile microfabricated platform to assess thermoelectric performance of diverse thin films, enabling sustainable energy harvesting solutions for IoT and autonomous devices.

## GRAPHICAL ABSTRACT

**Figure:**
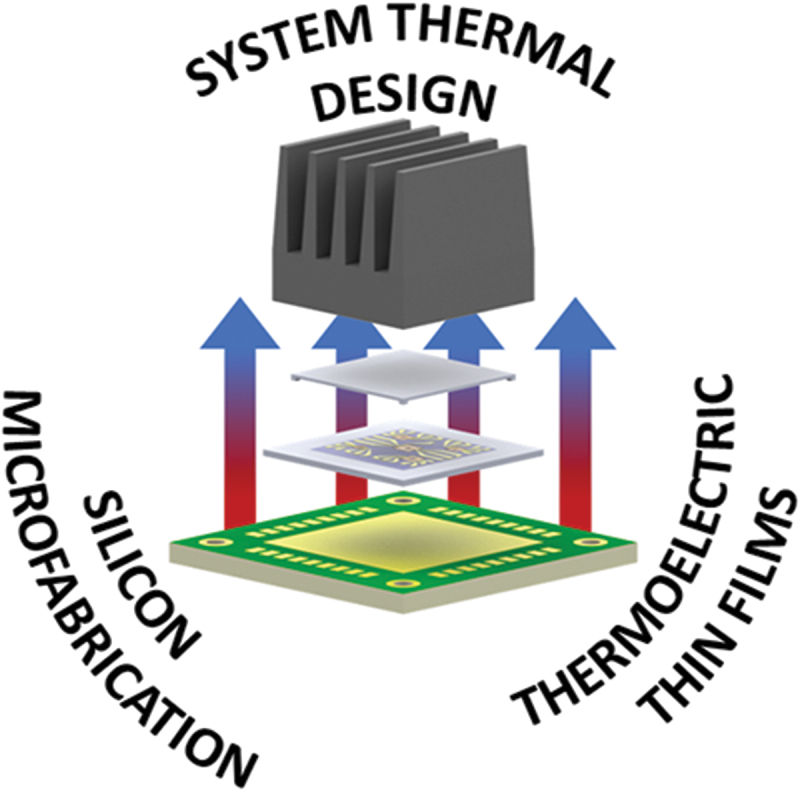

## Introduction

Thermal sources such as automotive engines, industrial processes, and home heating generate enormous amounts of waste heat [1] that could be converted into useful energy if efficient low-temperature thermal energy harvesters were available [2–4]. In this frame, thermoelectric generators (TEGs), solid state devices that convert heat (some temperature difference) into electric energy, become an attractive solution to self-powered electronic systems and as a sustainable alternative to conventional batteries, reducing the reliance on critical raw materials (CRM) in energy-related technologies. TEGs are of particular interest to power devices that are delocalised, or whenever accessibility to replace the batteries is challenging, for example, in Internet of Things (IoT) networks and harsh environments, such as industrial reactors, power stations, and volcanoes [5–10].

The thermoelectric efficiency of a material is expressed by the dimensionless figure of merit *zT*:

(1) $$\mathrm{zT}=\frac{S^{2}\sigma }{\kappa }T$$

where *S* is the Seebeck coefficient, *σ* is the electrical conductivity, *κ* is the thermal conductivity, and *T* is the temperature. The materials with the highest *zT* in the low and intermediate temperature ranges (from 300 K to 600 K [9]) are typically based on scarce and toxic elements, such as tellurium and lead. In addition, the fabrication of the related TEG faces limitations for mass production and miniaturization that hinder integration and deployment with mass-produced electronic devices like the nodes in IoT networks [11]. Consequently, current research in thermoelectric materials focuses on balancing performance with sustainability using more abundant and low-toxicity materials such as Half-Heusler alloys, lead-free chalcogenides, silicides, perovskites, and organic thermoelectrics [12], on novel technological solutions for the TEGs to better conform to the heat surfaces to optimise thermal efficiency at the device level and to unlock new uses, and on enabling mass production.

In the latter case, TEG technologies based on a modest *zT* material like Si benefit from miniaturisation, scalability, and efficiency in the use of materials provided by semiconductor micro- and nanotechnologies (MNTs) [10,13–15]. MNTs enable optimising the architecture of the micro-TEG (µTEG) and implementing thermal engineering to maximise the temperature difference across the thermoelectric material that critically determine the device performance, *ZT* [16–19]. It has been demonstrated how this approach can provide output powers well within the range of IoT needs [20–23].

The thermoelectric efficiency of a TEG is expressed by the dimensionless figure of merit *ZT*:

(2) $$\;\mathrm{ZT}=\frac{S^{2}G}{K}T$$

where *G* is the electrical conductance, and *K* is the thermal conductance, and *T* is the temperature. Here, it needs to be noted that *ZT* [17] is critically determined by the device architecture and thermal engineering at device level, which serve at maximizing the temperature difference across the thermoelectric material.

In this work, we demonstrate a multiscale approach to explore the integration of novel thin-film thermoelectric materials with Si-based micro-electromechanical systems (MEMS) technologies, to evaluate the impact of the related planar µTEG architecture (dimensions and shape of the active material) and device thermal management (heat dissipator integration with >100x extracted power improvement), and to benchmark their performance. As a proof-of-concept, 6 mol% niobium-doped strontium titanate (Nb:STO) thin films are patterned on square-shaped SiO_2_/Si_3_N_4_ planar membrane devices, a heat dissipator is implemented, and the performance of the related µTEG is evaluated.

## Nb:STO thin films and integration

SrTiO_3_ (STO)-based thin films offer a large variety of physical properties, such as metal-insulator transition, ferro-, piezo-, pyro-, and flexo-electricity, high thermopower, ferromagnetism, superconductivity, high electron mobility at low temperatures and resistive switching [24–27] making STO the ‘work horse’ of many oxide-based devices. It is well-known that Nb-doping in Ti-site of pristine insulating SrTiO3 (Nb:STO) makes it an n-type conductor, which exhibits good thermoelectric properties [28,29]. The highest power factor (*PF* = σ·S^*2*^) of ≈2.6 mW·m^−1^·K^−2^ was achieved near room temperature for STO films which are doped between 5% and 8% Nb [30,31].

In this work, Nb:STO thin films were grown by Pulsed Laser Deposition (PLD) with the same conditions previously reported in [32]. Structural analysis of the as-grown films was carried out by standard X-ray diffraction in a 2θ/ω configuration using Panalytical X′pert pro-MRD diffractometer (PANalytical, Netherlands). The thicknesses of the films were determined by deposition time calibration combined with using physical etching (RIE) and profilometer.

## µTEG Device Architecture

**Figure 1.**
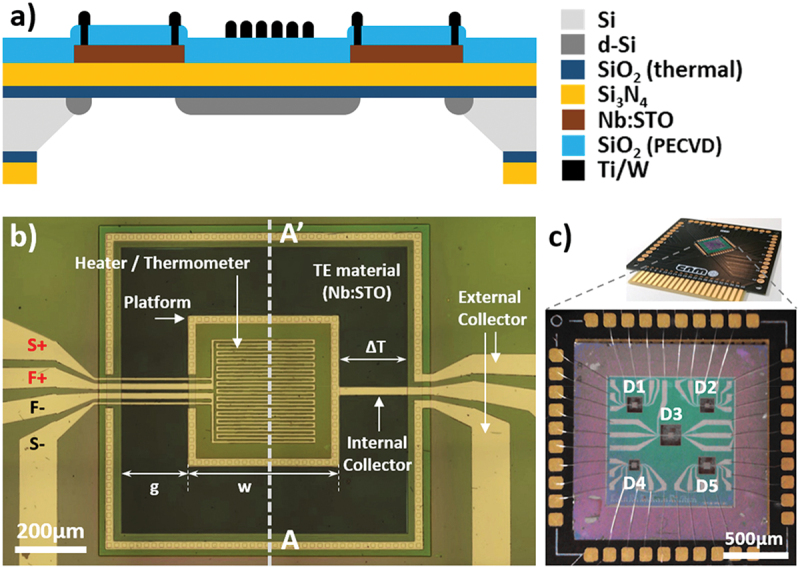
(a) schematic cross-section of the µTEG device corresponding to A-A’ in (b). b) optical top view microscope image of a single device (D4). (c) picture of the 1.5 × 1.5 cm^2^ chip mounted on a PCB for thermoelectric characterisation (top) and optical microscope image of a wire-bonded chip showing the five different devices (bottom).

The µTEG is a planar device that induces a temperature gradient across the thermoelectric material lying on a membrane (Figure 1). A doped silicon (d-Si) platform in the central part of the membrane hosts a metal serpentine that operates as a heater and thermometer, and homogenises the temperature. The serpentine is connected with a 4-wire scheme (force wires: F+ and F-, and sense wires: S+ and S-) to avoid contact resistance during resistance measurement. Current collectors contact the thermoelectric material at the edge of the d-Si platform (internal collector) and over the bulk silicon (external collector). The external current collector has two metal tracks that connect at the external contact pads. The chip hosts five devices (D1 to D5) with geometrical variations in the lateral length of the internal platform (*w*) and in the gap between the platform and the bulk Si (*g*) (Table 1).

**Table 1.** Geometric parameters for each of the five different devices on the chip.

	D1	D2	D3	D4	D5
*w* [µm]	400	300	400	400	500
*g* [µm]	400	400	600	200	400
*Area* = (*w* +2*g*)^2^ [mm^2^]	1.44	1.21	2.56	0.64	1.69

The architecture of the devices allows the evaluation of the performance of the µTEG in two different modes. In *test* mode, a thermal gradient is generated across the TE material by forcing a controlled current to the serpentine on the d-Si platform, which acts as a heater, and maintaining the bulk Si at room temperature. In *harvest* mode, the thermal gradient is naturally generated by placing the Printed Circuit Board (PCB) where the chip is mounted on a hotplate. The serpentine on the d-Si platform is used as a thermometer to evaluate the thermal gradient. The current on the thermometer is chosen to avoid Joule heating. This *harvest* mode is closer to an actual application, while the *test* mode allows evaluating the maximum possible performance under a given thermal gradient. In both cases, the heater can be used as a thermometer once its Temperature Coefficient of Resistance (*TCR*) has been characterized.

### Fabrication

The micro-fabrication of the µTEG is divided into two blocks. The first block comprises the main process steps to fabricate the platforms. It is performed at a wafer-scale level. The second block focuses on the integration of the thermoelectric Nb:STO thin film into the µTEG, and it is completed at chip-level because of sample size limitations at the PLD system.

Within the first block, the Si is selectively doped with boron to concentrations in the order of 10^20^ at cm^−3^ to define the d-Si platforms and the contour of the membrane areas. Next, 100 nm SiO_2_ are thermally grown and 300 nm Si_3_N_4_ are deposited by Low Pressure Chemical Vapor Deposition (LPCVD) on both sides of the wafer. This bilayer will form the membrane where the TE oxide material is to be deposited. Then, windows are patterned on the backside of the wafers by optical lithography and RIE, and a partial KOH etching of the Si is performed to leave 150 μm of bulk Si under the platform for structural robustness. At this point, the wafer is diced into chips.

Within the second block, the Nb:STO thermoelectric layer is first deposited by PLD. The thickness of the Nb:STO thin film layer (*t*_*Nb:STO*_) on the chip reported here is 149 nm. Then, the Nb:STO thin film is patterned by optical lithography followed by a dry etching process with Ar plasma. Next, a 200 nm thick SiO_2_ layer is deposited by Plasma Enhanced Chemical Vapor Deposition (PECVD) to serve as an interlevel dielectric and contacts to the Nb:STO are opened by optical lithography and wet etching. The metal layer is defined by an optical lithography, sputtering of 30 nm of Ti plus 200 nm of W and a lift-off process. Finally, the exposed Si on the backside is chemically etched with KOH to release the membranes. (see Figure S1 with steps detailing fabrication process).

The chip includes a set of electrical test structures (see Figure S2) to evaluate electrical technological parameters like the sheet resistance (Van der Pauw test structures) and the contact resistance between conducting layers (Kelvin test structure). The electrical conductivity is calculated based on the sheet resistance measurements and the thickness of the layer (*σ* = 1/(*R*_*□*_∙*t*)). The sheet resistance obtained for the Nb:STO is *R*_*□,Nb:STO*_ = 49 kΩ and the calculated electrical conductivity is *σ*_*Nb:STO*_ = 1.37 Ω^−1^·cm^−1^. The contact resistance between metal and Nb:STO is *R*_*c*_ = 6.8·10^−3^ Ω·cm^2^ on a 20 × 20 µm^2^ contact area (see Table S1).

### Characterization

Before evaluating the performance of the device in *test* or *harvest* modes, the heaters in the centre of each platform need to be calibrated by measuring their temperature coefficient of resistance (*TCR*) to use them as thermometers. To calculate the thermal gradient seen by the thermoelectric material, the temperature of the bulk Si was measured on similar setups. It was determined to be the same temperature as that of the bottom PCB within ±0.5°C.

*TCR* measurements were made on a PCB with the wire-bonded device inside an oven. Before any measurement was made, the highest possible current at which the heater temperature remained stable and unaffected by Joule heating was determined. As an additional test, the open-circuit output voltage of the device, *V*_*OC*_, was also evaluated at different currents on the heater to validate that they would not generate a temperature gradient. It was found that 50 µA was the highest current that did not generate a measurable *V*_*OC*_ in any of the devices on the chip. The resistance of the heaters was measured with this current at six different temperatures, ranging from room temperature (RT) to 175°C. Then, the *TCR* value for each of the heaters can be obtained using Equation (3):

(3) $$R=R_{0}\cdot \left(1+\mathrm{TCR}\cdot \left(T-T_{0}\right)\right)$$

where *R*_*0*_ is the value of the resistance at *T = T*_*0*_, and *T*_*0*_ is the room temperature.

In addition to the thermoelectric characterization, a dedicated chip was used to measure the in-plane thermal conductivity by the 3ω Völklein method [17]. The obtained value for a 25-nm-thick sample of Nb:STO was *k*_*Nb:STO*_ = 3.118 W·m^−1^·K^−1^ at room temperature. The obtained value is lower than bulk STO due to phonon boundary scattering in thin films and the additional point-defect scattering introduced by Nb doping,

## Device performance and discussion

**Figure 2.**
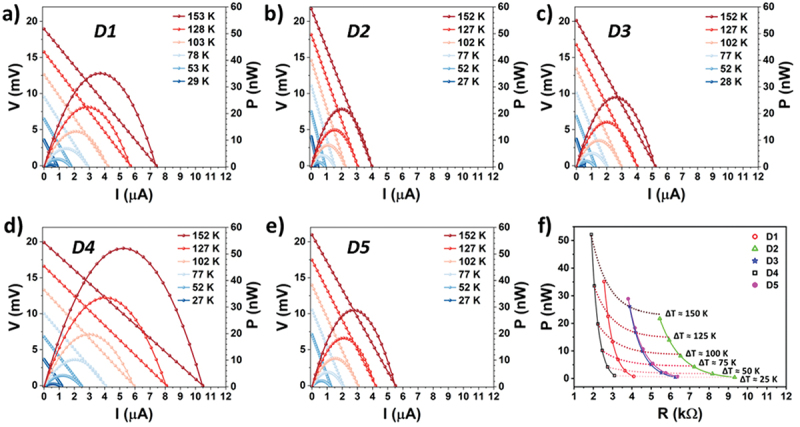
(a-e) *test* mode performance plots for the five devices exposed (D1 to D5) to different *∆T*. (f) influence of electric resistance on the maximum power generated by the five devices at different *∆T*. All *∆t* reported are relative to room temperature *T_amb_* =25°C.

The device performance was evaluated by applying current to the heater (*test* mode). Adjusting the current makes it possible to control the temperature of the central part of the membrane, while the temperature of the cold side was measured with a thermocouple on the bottom of the PCB. For each *ΔT* across the device, a performance plot (voltage and power vs. current) was obtained. Figure 2(a–e) shows the performance plots for the five devices on the chip at different *∆T*. The maximum power (*P*_*max*_) obtained was for the D4 device with a total of 53.26 nW at *∆T* = 152 K. The maximum power density (*P*_*dens*_ = *P*_*max*_*/Area*) resulted in 8.32 µW·cm^−2^. To compare with other devices at different temperature gradients, it is useful to report the specific power density as defined in [11] which is *Γ* = 0.36 nW·cm^−2^·K^−2^. From the slope of the *I-V* curves shown in Figure 2, it is possible to calculate the electrical resistance (*R*) of the µTEG, which in this case is mainly dominated by the thermoelectric material. Then, plotting the maximum power for each device at different *∆T* versus the electrical resistance (see Figure 2(f)), it becomes clear the importance of a small electrical resistance in a power generator. The highest output power was obtained for device D4, which has the smallest electrical resistance.

From the slope of the *V*_*OC*_ vs *ΔT* curves in Figure 2, we calculated an average Seebeck coefficient of *S* = −135.7 ± 8.4 µV·K^−1^ (see Figure S3). The thermal conductance (*K*) defined as the ratio between the heater power and the temperature gradient obtained was calculated from the slope of the heater power vs *ΔT* curve, giving values from 62 to 105 µW·K^−1^ depending on each device (see Figure S4). Using data from *harvest* and *test* modes, the thermal conductance of the device K_TEG_ can be obtained (see S5 model and discussion).

**Figure 3.**
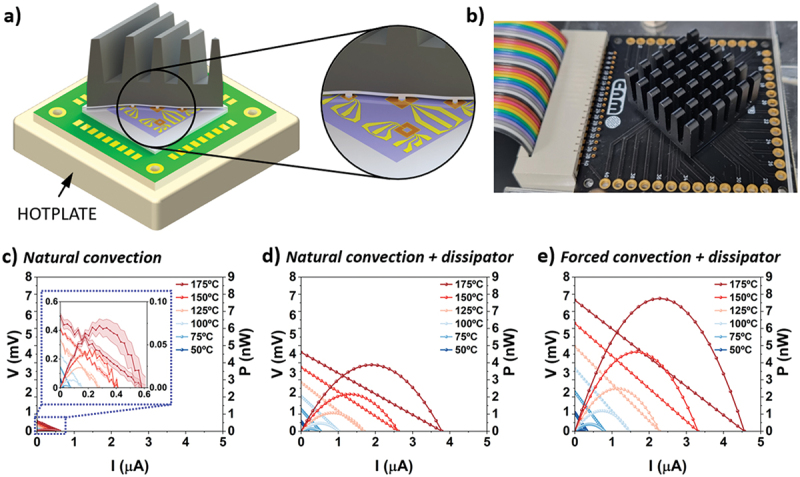
(a) scheme of the dissipator mounting strategy by using an intermediate adapter for harvest mode measurements and placing the PCB on top of a hotplate. (b) image of the PCB containing the chip with adapter and dissipator on top. Performance plots in *harvest* mode for the device D4 with different thermal management configurations, with hot-plate temperatures from 50°C to 175°C, under (c) natural convection, (d) natural convection with dissipator, and e) forced convection with dissipator.

Each device was also measured in *harvest* mode, by placing the PCB on a hotplate, with temperatures ranging from 50 to 175°C. As shown in Figure 3(c), the *V*_*OC*_ measured in *harvest* mode was significantly lower than in *test* mode. This discrepancy is due to the large thermal resistance of the central part of the membrane and the ambient under natural convection conditions. Since thermal resistance is inversely proportional to the surface area, smaller areas result in greater thermal resistance. For example, when the device D4 was placed on a hotplate at 175°C, the central part of the membrane reached 151°C, yielding a temperature gradient of 24°C across the TE material —16% of the total gradient between the hotplate and room temperature. This thermal bottleneck is irrelevant in *test* mode, where the platform temperature is directly controlled via a heater (see Figure S5 and discussion).

Optimizing thermoelectric harvesting devices requires a multiscale design approach – enhancing not only material properties but also device architecture and system-level thermal management. To improve the performance of the µTEG, a heat dissipator was mounted on top of the chip, as shown in Figures 3(a) and 3(b). For effective operation, the dissipator must contact only the central regions of the membranes, thereby minimizing thermal resistance to the ambient. To achieve this selective contact, we designed and micromachined custom Si adapters that support the dissipator while minimizing thermal coupling with the hot regions of the device. A similar strategy was previously reported in reference [21]. These adapters consisted of 300-µm-thick Si square plates with a smaller lateral dimension than the chip, each equipped with five pillars located on top of each of the central membranes. Additionally, four corner pillars 200 µm thick, that act as spacers, were included to support the weight of the dissipator and prevent any damage to the membranes.

The dissipator resulted in a 61-fold increase in power output compared to the setup without this thermal management element (*P*_*max*_ = 0.07 nW vs *P*_*max*_ = 4.28 nW for *T*_*HP*_ = 175 ºC), as shown in Figure 3(c–d)). The power output increase reached a factor ca. 110 with the dissipator and under forced air (1.7 m·s^−1^) convection (*P*_*max*_ = 7.75 nW for *T*_*HP*_ = 175°C, see Figure 3(e)). While thermal management with a dissipator and forced convection provided better results, the power generated in *harvest* mode using this device architecture still remains approximately seven times lower than in *test* mode, highlighting the importance of thermal management at device level.

**Figure 4.**
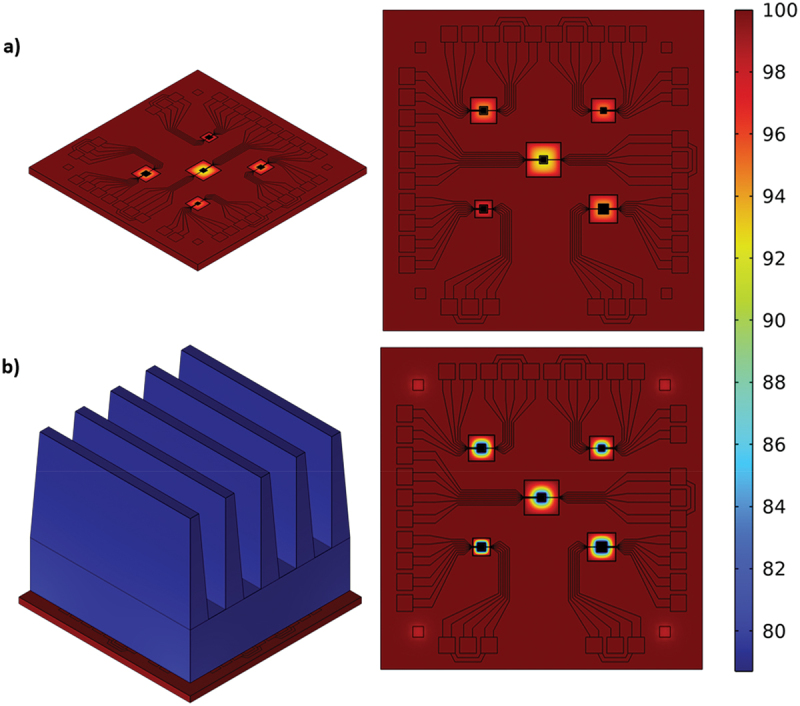
Temperature (in ºC) results of the finite element model (COMSOL) corresponding to (a) the µTEG in natural convection with heat exchange coefficient *h* = 10 W·m^−2^·K^−1^ and (b) the µTEG with adapter and heat dissipator under forced convection with *h* = 20 W·m^−2^·K^−1^, corresponding to an air velocity of 1.7 m·s^−1^. a temperature of *T_HP_* =100°C is set at the bottom of the model to account for the hotplate temperature and *T_amb_* =25°C.

A finite element model has been developed to identify possible improvements and compare with experimental results. Figure 4 shows two cases: a) the µTEG under natural convection; and b) the µTEG with adapter and dissipator under a forced convection corresponding to air at 1.7 m·s^−1^. The heat exchange coefficient, which defines how much heat is exchanged with the ambient, is set at *h* = 10 W·m^−2^·K^−1^ for the natural convection case and *h* = 20 W·m^−2^·K^−1^ for the forced convection case. The adapter and dissipator are hidden in the left image of b) to compare the temperature map for both configurations. Both models have a Dirichlet boundary condition applied at the bottom of the µTEG corresponding to the hotplate temperature and set at *T*_*HP*_ = 100°C, and the air temperature surrounding the device is set at *T*_*amb*_ = 25°C. A thin layer of thermal paste has been added on the contact surfaces between the adapter and the µTEG (the four corners and five membranes) with a thickness of 10 μm and a thermal conductivity of 3 W·m^−1^·K^−1^ (no pressure is applied in the adapter and dissipator assembly, to minimize the risk of membrane collapse). The maximum power obtained for the D4 device in the model is *P*_*max*_ = 5.29pW for the µTEG alone, and *P*_*max*_ = 360.36pW for the µTEG with adapter and dissipator, resulting in a 68-fold improvement. While the match with experimental results is not quantitative, a similar improvement is obtained when comparing both thermal management options. The main inaccuracies of the model are the convection boundary conditions and the thermal contact between adapter and µTEG. In addition, it has been identified that some parasitic heat-flow paths form the four corner pillars reach the membrane, reducing its performance. Efforts are now being taken to modify the adapter design, especially the corner pillars (combining low thermally conducting ceramic materials with silicon microfabricated parts, and reducing the footprint) to enhance the power output.

Combining the different results obtained from electrical conductivity (*σ*_*Nb:STO*_ = 1.37 Ω^−1^·cm^−1^), thermal conductivity (*κ*_*Nb:STO*_ = 3.118 W·m^−1^·K^−1^) and average Seebeck coefficient (*S*_*Nb:STO*_ = −135.7 µV·K^−1^), we can compute the material power factor and *zT*, resulting in *PF* =2.52 µW·m^−1^·K^−2^ and *zT* =2.43·10^−4^ at *T* =300 K. Similar calculations can be done for the device *ZT*. In this case, if we focus on the best performing device (D4), we need the electrical conductance (*G*_*D4*_ = *1/R*_*D4*_ = 3.23·10^−4^ Ω^−1^), the thermal conductance (*K*_*TEG,D4*_ = 79.37 µW·K^−1^, see S5) and the Seebeck coefficient (*S*_*D4*_ = −143.2 µV·K^−1^, see Fig S3). The calculated value for *T* = 300 K is *ZT* = 2.50·10^−5^. These results are significantly lower than the state-of-the-art values reported for Nb:STO. While both the thermal conductivity and Seebeck coefficient are consistent with previously reported values, the electrical conductivity measured in our device (as listed in Table 1) is substantially lower than the values reported in the literature for 6% Nb-doped STO [31,33]. This discrepancy may be attributed to the fact that most reported values for Nb:STO are obtained from films grown on lanthanum strontium aluminium tantalate (LSAT) substrates and other crystalline substrates that promote crystalline and epitaxial growth. In contrast, in our case, Nb:STO was deposited onto an amorphous substrate, namely LPCVD-SiN_x_, resulting in a non-epitaxial and polycrystalline film. Consequently, its functional properties are degraded due to the distinct microstructural characteristics induced by growth on an amorphous substrate, as opposed to those achieved on substrates optimized for epitaxy and crystallinity. With the integration of appropriate buffer layers (like TiO_2_ [34–36] or CeO2 [37,38]), the electrical conductivity of our TE thin film could potentially match the reported value of 800 S·cm^−1^ [32]— corresponding to a 149 nm-thick 6% Nb:STO layer at 300 K. Based on our measured Seebeck coefficient and thermal resistance, this would translate into a power factor of *S*^*2*^*·σ* = 1.47 mW·m^−1^·K^−2^, yielding a *zT* = 0.142 and *ZT* = 1.46·10^−2^ at 300 K.

A final comparison with state-of-the art references has been included in Table 2 for the most relevant parameters in terms of µTEG performance. Data from our D4 device are also included for comparison, and a D4* device where the electrical conductivity could reach the 800 S·cm^−1^ value. The table shows the relevance of the internal resistance which has a huge impact on the final *Pmax* obtained, as well as the impact of a proper architecture which facilitates the heat extraction in microstructures reflected in the final *ΔT* seen by the thermoelectric material.

**Table 2.** Comparison with state-of-the-art references with D4 device for most relevant parameters for µTEG performance.

Ref	Γ μW·cm^−2^·K^−2^	P_dens_ μW·cm^−2^	R_int_ Ω	zT/ZT	footprint cm^2^	∆T (condition) K
[11]	**0.052**	**1.3**	**52.8e6**	-/-	**1**	**5 (forced)**
[21]	**6.4e-3**	*67.45*	*51.04*	-/-	**0.49**	**49.5 (T**_**HP**_ **=200ºC)**
[22]	**1.3**	**100**	**38.8**	**0.02/0.016**	**0.077**	**10 (RT + 10ºC)**
D4	**3.6e-4**	**1.21**	**3096**	**2.4e-4/2.5e-5**	**6.4e-3**	*46.1* *(T*_*HP*_ *=175ºC)*
D4*	*0.21*	*707.1*	*5.302*	*0.142/0.0146*	**6.4e-3**	*46.1* *(T*_*HP*_ *=175ºC)*

Where available, *ZT* and *zT* are evaluated at 300K. All values found in the references are in bold, while those in italics are calculated from data in the references. D4* corresponds to the D4 device assuming an electrical conductivity of 800 S·cm^−1^ instead of the obtained one of 1.37 S·cm^−1^.

## Conclusions

Most efforts in the thermoelectric community are focused on optimizing the material *zT*, and very few are dedicated to thermal management at device (architecture) and system level to improve *ZT*. In contrast, this work focuses on developing a versatile test platform to evaluate almost any thermoelectric material in thin film form under real operating conditions. While the obtained results do not show an alternative to state-of-the-art thermoelectric devices, they prove the platform flexibility to evaluate the integration of thin film Nb:STO and to evaluate its performance as a µTEG. To the authors knowledge, this work is the first reporting a thin film Nb:STO-based µTEG.

## Supplementary Material

Supplemental Material
