# Supplementary material for: A novel approach to micro-fabricated thermoelectric generators with SrTiO3
Source: Sci Technol Adv Mater. 2026 May 8;27(1):2665920. doi: 10.1080/14686996.2026.2665920 (PMC13237791; doi:10.1080/14686996.2026.2665920)
Supplement: Supplemental Material [file TSTA_A_2665920_SM1412.docx]

Supplementary Information

A novel approach to micro-fabricated thermoelectric generators with SrTiO_3_

Joaquin Santander^a^, Iñigo Martin-Fernandez^a^, Carlos Carbonell^a^, Alex Rodríguez-Iglesias^a^, Laura Fuentes-Rodríguez^a^, Marta Fernández-Regúlez^a^, Llibertat Abad^a^, Aitor F. Lopeandia^b,c^, Arindom Chatterjee^d^, Nini Pryds^d^, Luis Fonseca^a^, and Marc Salleras^a*^

^a^Institute of Microelectronics of Barcelona (IMB-CNM-CSIC), 08193 Bellaterra, Spain.

^b^Physics Department, Universitat Autònoma de Barcelona, 08193 Bellaterra, Spain.

^c^GTNAM at Institut Català de Nanociència i Nanotecnologia (ICN2), 08193 Bellaterra, Spain.

^d^Department of Energy Conversion and Storage, Technical University of Denmark, 2800 Kgs Lyngby, Denmark.

*marc.salleras@csic.es


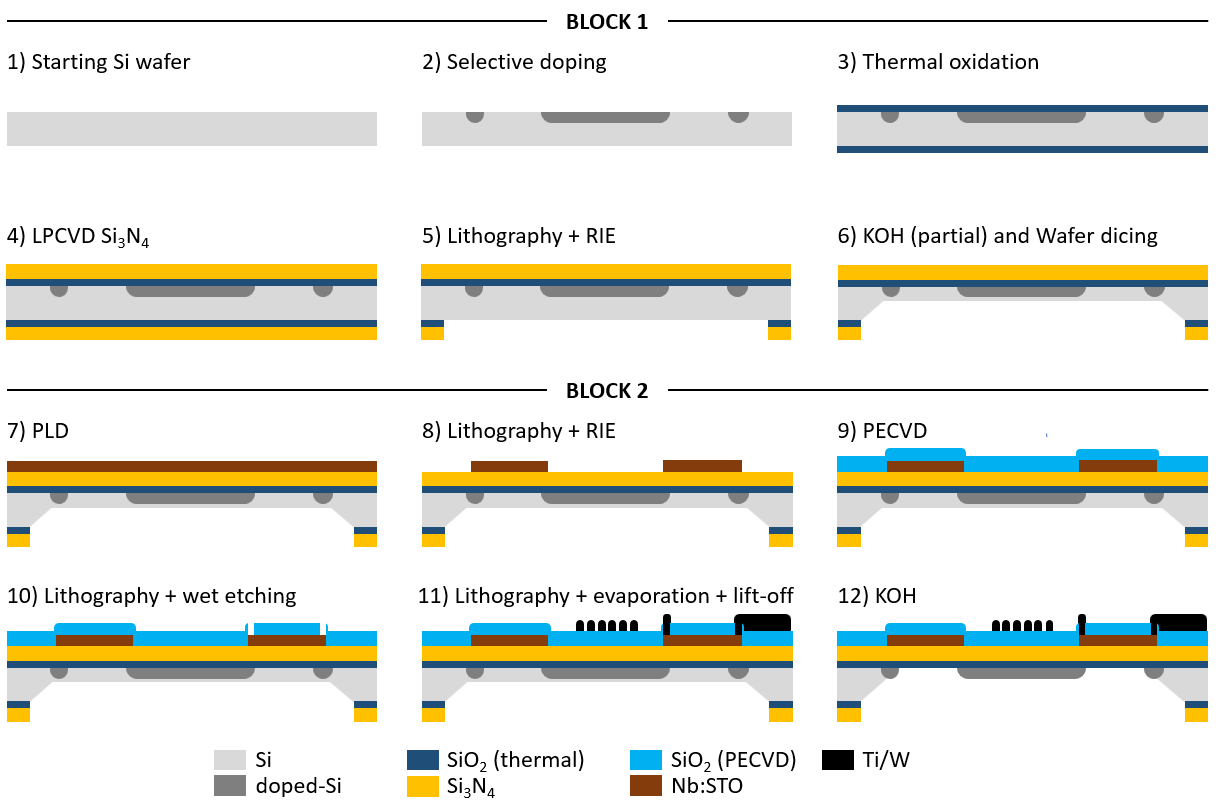
 **Figure S1**: Schematics of the fabrication scheme. The fabrication is divided in 2 blocks. Block 1 comprises the process steps at a wafer-scale level. Block 2 comprises the process steps at a chip-scale level. (1) Starting wafers; (2) Selective doping of Si with BBr_3_; (3) Growth of 100 nm SiO_2_ by thermal oxidation (4) deposition of low stress Si_3_N_4_ by LPCVD; (5) Optical lithography followed by RIE and photoresist removal to pattern the membrane windows; (6) Partial wet etch with KOH of the Si and wafer dicing into chips; (7) PLD deposition of the Nb:STO; (8) Optical lithography followed by Ar plasma and photoresist removal to pattern the Nb:STO; (9) PECVD deposition of 200 nm SiO_2_ as interlevel oxide; (10) Optical lithography, wet etching, and photoresist removal to define the bias to the Nb:STO; (11) Optical lithography, evaporation of 10 nm Ti and 200 nm W and lift-off to pattern the current collectors and the heaters, the tracks and the metal pads; and (12) Complete KOH etching of the Si to define the membranes.


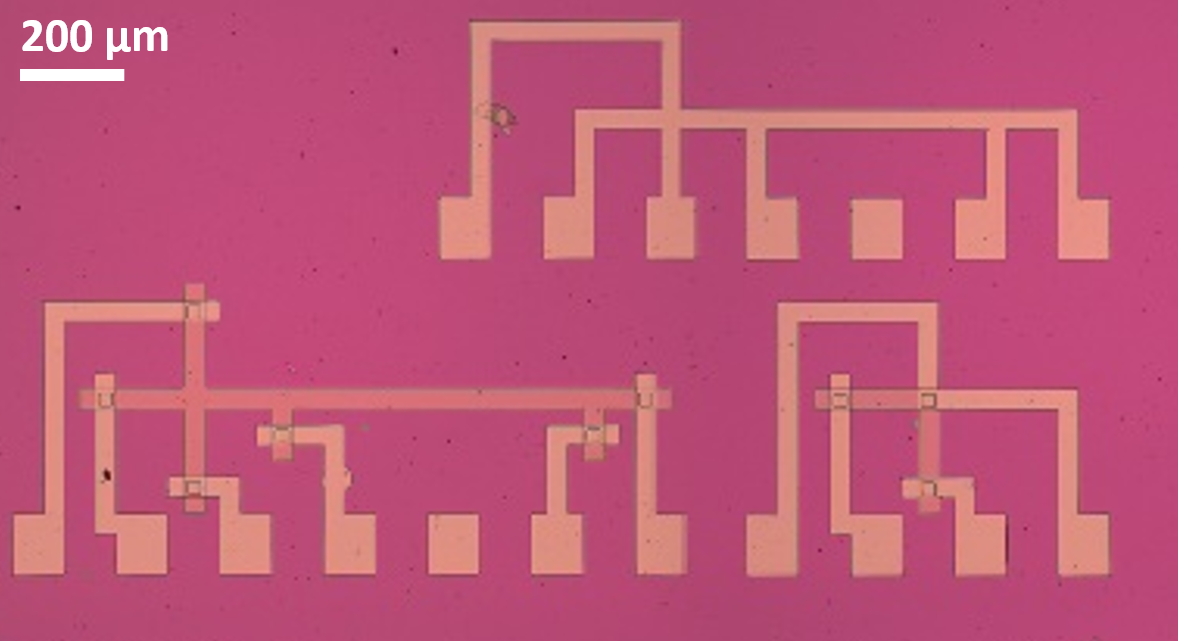


**Figure S2.** Test structures used for the measurement of electrical parameters.

The chips include a set of electrical test structures able to measure basic electrical technological parameters like the sheet resistance of conducting layers (Van der Pauw test structures, lower left image in Figure S2) and the contact resistance between conducting layers (Kelvin test structure, lower right image in Figure S2). Table S1 summarizes the obtained values, which confirm that the chips were fabricated as expected. The value of the sheet resistance of the Nb:STO permits the calculation of the electrical conductance provided the thickness of the layer (149 nm).

**Table S1**. Measurements obtained for the technological parameters: sheet resistances of the metal and of the Nb:STO layers and contact resistance between the metal and the Nb:STO layers in a 20 x 20 µm^2^ contact area.

| R_□_ metal | 1.3 Ω/□ |
| --- | --- |
| R_□_ Nb:STO | 49 kΩ/□ |
| R_c_ metal to Nb:STO | 1.7 kΩ |


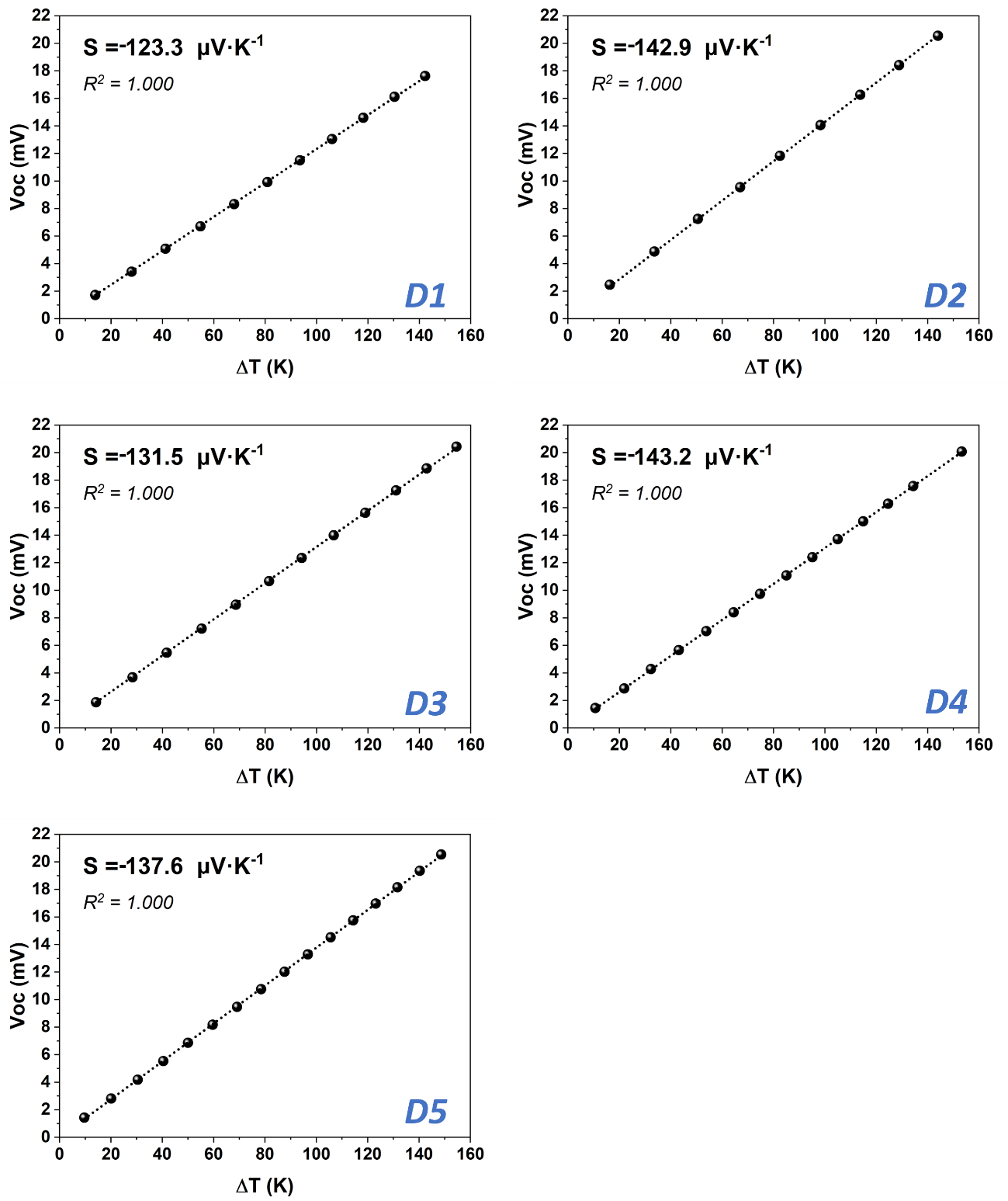


**Figure S3.** Determination of the Seebeck coefficient (*S*) for each of the five devices from the slope of the *V_OC_* vs *ΔT* curves. The average is *S* = -135.7 ± 8.4 μV·K^-1^.


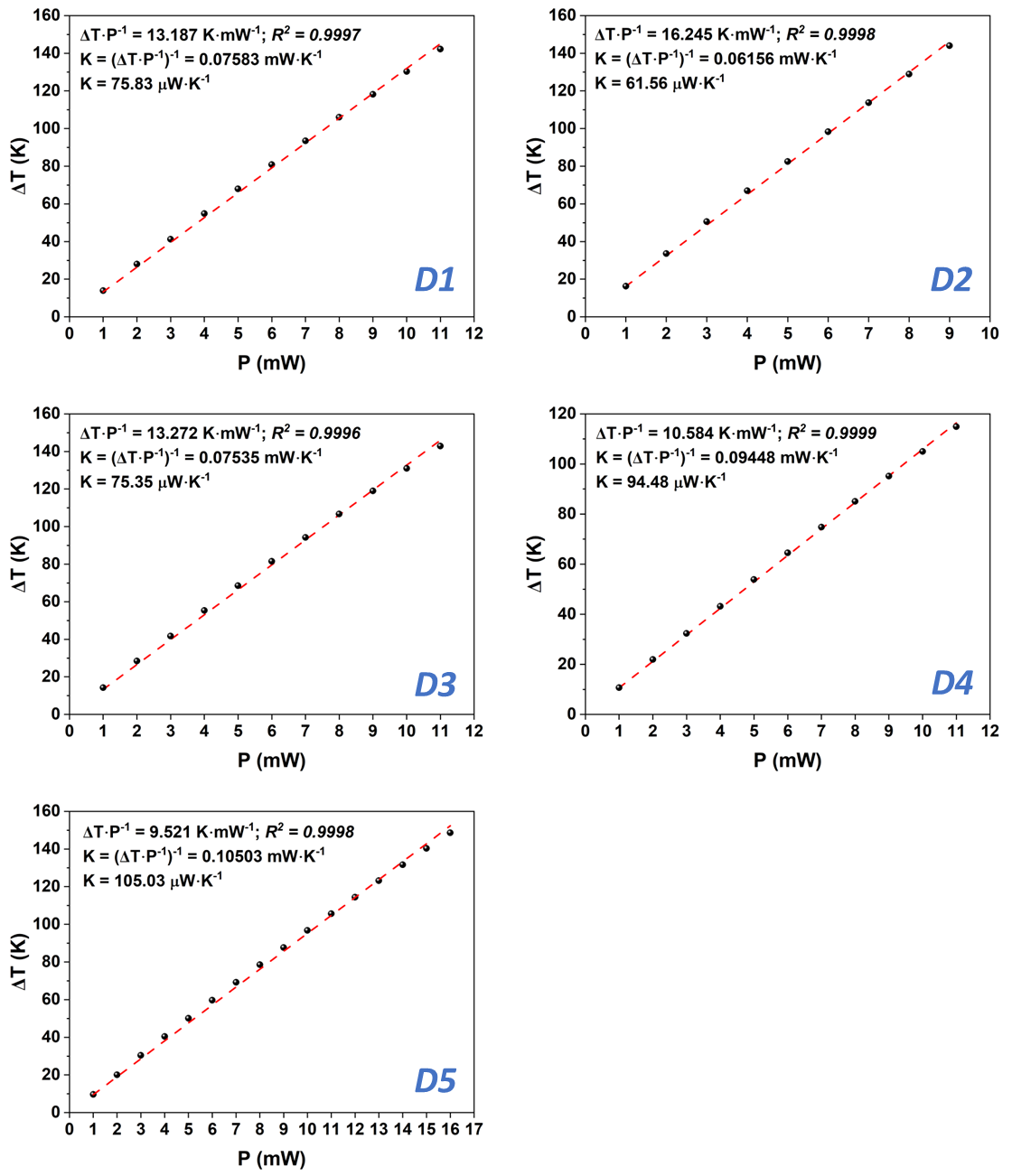


**Figure S4.** Determination of the thermal conductance (*K*) of the five devices from the slope of the *ΔT* vs heater power curves.


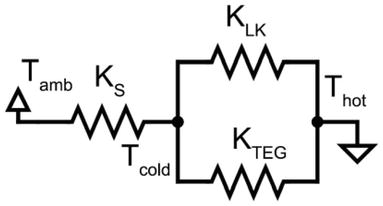


**Figure S5:** Simplified thermal model of the µTEG.

In the calculation of the thermal conductance of the TEG (*K_TEG_*) the simple thermal model in Figure S5 is used. In this model the node *T_cold_* is where the heater/thermometer is placed and *T_hot_* is the hotplate. *K_TEG_* is the thermal conductance of the device (the thermoelectric membrane), *K_LK_* is the leakage thermal conductance (through parasitic heat-flow paths), and *K_S_* is the thermal conductance from the heater/thermometer towards the ambient.

For this discussion, we will focus on D4 device and will ignore the *K_LK_* term as it is expected to be much smaller than *K_TEG_*. From our *test* mode results, we can calculate the thermal conductance from the slope of *ΔT* vs heater power curves (Figure S4) resulting in *K_D4_* = 94.48·μW·K^-1^. According to the thermal model, this thermal conductance corresponds to the parallel connection of *K_TEG_* and *K_S_*, therefore *K_D4_* *= K_TEG,D4_ + K_S,D4_*. In *harvest* mode we set the hotplate temperature at 175 ºC, and the heater (used as a thermometer) measures a temperature of 151 ºC, while the ambient temperature measured is 25 ºC. Then we can calculate for D4:

$$Q\text{=}dT\cdot K\to Q\text{=}\left( 175\text{-}151\text{ }℃ \right)\cdot K_{TEG}\text{= }\left( 151\text{-}25\text{ }℃ \right)\cdot K_{S}$$

$$24\text{ }℃\cdot K_{TEG}\text{= }126\text{ }℃\cdot K_{S}\to K_{TEG}\text{=}{126}/{24}\cdot K_{S}\text{= }5.25\cdot K_{S}\text{ }$$

And using: $K_{TEG}\text{+}K_{S}\text{=}94.48\cdot\text{ }\text{μ}W\cdot K^{-1}$

We can obtain: $K_{TEG}\text{=}79.37\cdot\text{μ}W\cdot K^{-1}\to K_{S}\text{=}15.12\cdot\text{μ}W\cdot K^{-1}$

The *K_TEG_* value obtained can be used to calculate a *ZT* value.
